# Mating Design and Genetic Structure of a Multi-Parent Advanced Generation Intercross (MAGIC) Population of Sorghum (*Sorghum bicolor* (L.) Moench)

**DOI:** 10.1534/g3.117.300248

**Published:** 2017-11-17

**Authors:** Patrick O. Ongom, Gebisa Ejeta

**Affiliations:** * Department of Agronomy, Purdue University, West Lafayette, Indiana 47907; † Center for Global Food Security, Purdue University, West Lafayette, Indiana 47907

**Keywords:** multi-parent advanced generation intercrosses (MAGIC), genotyping-by-sequencing (GBS), population structure, single nucleotide polymorphisms (SNPs), linkage disequilibrium (LD), MPP

## Abstract

Multi-parent advanced generation intercross (MAGIC) populations are powerful next-generation mapping resources. We describe here the mating design and structure of the first MAGIC population in sorghum, and test its utility for mapping. The population was developed by intercrossing 19 diverse founder lines through a series of paired crosses with a genetic male sterile (MS) source, followed by 10 generations of random mating. At the final stage of random mating, 1000 random fertile plants in the population were identified and subjected to six generations of selfing to produce 1000 immortal MAGIC inbred lines. The development of this sorghum MAGIC population took over 15 yr. Genotyping-by-sequencing (GBS) of a subset of 200 MAGIC lines identified 79,728 SNPs, spanning high gene-rich regions. Proportion of SNPs per chromosome ranged from 6 to 15%. Structure analyses produced no evidence of population stratification, portraying the desirability of this population for genome-wide association studies (GWAS). The 19 founders formed three clusters, each with considerable genetic diversity. Further analysis showed that 73% of founder alleles segregated in the MAGIC population. Linkage disequilibrium (LD) patterns depicted the MAGIC population to be highly recombined, with LD decaying to *r*^2^ ≤ 0.2 at 40 kb and down to *r*^2^ ≤ 0.1 at 220 kb. GWAS detected two known plant height genes, *DWARF1* (chromosome 9) and *DWARF3* (chromosome 7), and a potentially new plant height quantitative trait locus (QTL) (*QTL-6*) on chromosome 6. The MAGIC population was found to be rich in allelic content with high fragmentation of its genome, making it fit for both gene mapping and effective marker-assisted breeding.

Quantitative traits in crop plants form the predominant plant characteristics of agronomic and economic importance, including such traits as crop productivity, environmental adaptation, and tolerance to drought, as well as resistance or immunity to certain pests and diseases. The inheritance of these traits is often complex, with patterns of phenotypic expression determined by an intricate series of interactions between multiple genes and environmental factors. Conventionally, allelic sources to improve these traits in crops are sought from naturally occurring variation (Kover *et al.* 2009), but the genetic underpinning of natural variation in these traits often appears ambiguous, partly due to lack of appropriate core genetic resources to properly exploit such variation. The more common way to dissect QTL in crop species has been through use of populations derived from biparental crosses, including F_2_, backcross, recombinant inbred lines, backcross inbred lines, double haploids, and near isogenic lines (Collard *et al.* 2005; Rakshit *et al.* 2012). Immortal inbred lines from biparental populations have been used for genetic studies and mapping of QTL linked to key traits in many crops, including sorghum, where putative loci for drought resistance (Tuinstra *et al.* 1997), cold tolerance (Knoll *et al.* 2008), *Striga* resistance (Vogler *et al.* 1996; Haussmann *et al.* 2004), and disease and insect resistances (Apotikar *et al.* 2011) have been identified.

With advances in genomic technologies that allow high-throughput genotyping capabilities in crops at a continuously reduced cost, alternative QTL mapping resources have recently emerged. These new advances have immensely increased the larger marker density required for QTL studies, thrusting plant scientists into the genomic era and embracing GWAS as a tool to dissect QTL underlying complex traits as well as to identify functional genes, which initially have been used only in human populations. The potential utility of GWAS for more precise mapping of novel genes in crop plants has been demonstrated by a number of studies (Gupta *et al.* 2005; Nordborg and Weigel 2008; Rafalski 2010). The mapping resources that have commonly been used for GWAS are accessions of landrace genetic resources or breeding lines that have been extensively phenotyped over years in most breeding programs. These collections have provided more cost-effective mapping panels that are diverse and have had high historical recombination that is suitable for GWAS. Furthermore, the faster LD decays in natural accessions make it possible to map QTL with higher resolution (Nordborg and Weigel 2008; Kover *et al.* 2009). However, natural accessions tend to be highly stratified, requiring the use of complex statistical models to account for the false association signals that result in GWAS conducted using them.

More recently, novel MAGIC populations have been advanced as next-generation mapping resources to address the major limitations of existing mapping populations (Bandillo *et al.* 2013; Mackay *et al.* 2014; Verbyla *et al.* 2014; Holland 2015; Huang *et al.* 2015). The mouse collaborative cross was the first MAGIC population to be developed before the approach was proposed for QTL mapping of complex traits in a crop plant (Cavanagh *et al.* 2008). The MAGIC design concept is similar to that of advanced intercross lines (AILs) proposed by Darvasi and Soller (1995), except that AILs are still derived from a two-parent cross; however, each generation is sequentially and randomly intercrossed until advanced intercross generations are attained. MAGIC populations are generally created by intercrossing multiple founder lines over several generations. Using multiple founders contributes more allelic diversity than that captured in typical biparental mapping populations. Multiple cycles of intercrossing result in greater opportunities for recombination, thus providing greater precision in QTL location. MAGIC and MAGIC-like populations have now been made available in a range of plant and crop species, including *Arabidopsis* (Kover *et al.* 2009), wheat (Huang *et al.* 2012; Mackay *et al.* 2014), rice (Bandillo *et al.* 2013), and maize (Dell’Acqua *et al.* 2015).

Multi-parent crosses have a long history in conventional plant breeding, especially in outcrossing crops where crosses are easy to make (Harlan and Martini 1929; Suneson 1956). In self-pollinated species like sorghum, multi-parent crosses gained usage in 1960s after the discovery of genetic male sterility (GMS) systems, which facilitated the adoption of recurrent selection (Eberhart 1972; Reddy and Stenhouse 1994). It is with the capacity to generate large populations more efficiently and recent tools in high-throughput genomic technologies, as well as in advanced statistical capabilities, that multi-parent populations have been employed in genetic mapping. Although MAGIC populations are useful in genetic studies, they present some unique challenges. The crossing schemes of the currently available MAGIC populations pose a potential intermating bias that can result in assortative mating other than the assumed random mating. Depending on the number of founders, intermating may become cumbersome as family sizes of the parent crosses increase along the pedigree. This complexity increases the chances of mating between individuals with similar genotypes, causing deviations from random mating expectation, a phenomenon termed assortative mating (Templeton 2006). Hence, it is often advised that staggered planting and/or planting of the same families multiple times should be avoided during MAGIC development, because these practices would promote assortative mating (Kole 2013). Assortative mating would introduce the rise of subgroupings within the MAGIC population, distorting LD and causing spurious associations (Fisher 1919; Moran *et al.* 1966; Wilson 1978; Garnier-Géré and Chikhi 2013), and bringing about the false associations often encountered in natural populations. Times required to establish MAGIC populations can be long, given the number of founders involved, making the process logistically challenging and labor intensive. In sorghum, the use of GMS in the construction of a MAGIC population significantly reduces the challenges described above.

The objectives of this study were as follows: (i) to describe the construction and mating design of the first sorghum MAGIC population facilitated with the aid of GMS in a base population, (ii) to construct a genome-wide single nucleotide polymorphism map and, utilizing these SNPs, to dissect the structure of the MAGIC population, and (iii) to evaluate the efficiency of the SNP panel and the potential of the sorghum MAGIC population for conducting GWAS using plant height, a trait that is well studied in sorghum, with some of the governing loci that have already been cloned.

## Materials and Methods

The MAGIC population was constructed from 19 founder sorghum lines that had previously been characterized for a broad range of useful agronomic traits, including early maturity, broad adaptation, yield potential, drought tolerance, and grain quality, as well as pest and disease resistance. The 19 founders were intermated and recombined with the aid of a GMS system that facilitated random mating for several cycles to produce 1000 random inbreds, through further selfing through a single-seed descent (SSD) method of inbreeding. A subset of 200 S_7_ inbred lines were pooled from the MAGIC population for use in this study. The 200 S_7_ inbreds were phenotyped for plant height at the Agronomy Center for Research and Education, Purdue University, during the summer of 2013 and 2014. Tissue from each genotype were collected, lyophilized, and pulverized for DNA extraction. Thirty-microliter aliquots of DNA at concentrations of 50–100 ng/µl in 96-well plates were sent to the genomic facility at Cornell University for genotyping using a high-throughput GBS platform. The reference genome was indexed and alignment generated with the software package BWA Version: 0.7.8-r455 (Li and Durbin 2009). Of the 4,122,598 tags used, 2,763,236 (67.0%) were aligned to unique positions, while 457,904 (11.1%) were aligned to multiple positions, and 901,458 (21.9%) could not be aligned. Chromosome-wide density plots for SNPs and the reference sequence genes were generated to gauge genomic coverage of the discovered SNPs. To determine the proportion of founder alleles that were captured in the MAGIC subset, the MAGIC and founder SNPs were filtered separately, allowing minor allele frequency (MAF) > 0.01 and 10% missing data, and the number of polymorphic SNPs in both panels for each chromosome were counted. The pattern of genetic structure of the MAGIC population was assessed using principal component analysis (PCA) and neighbor-joining tree analyses. PCA scores were generated in TASSEL and exported to Microsoft Excel, where visualization graphics were produced. To evaluate the effectiveness of the sorghum MAGIC population for use in gene mapping, data on plant height was subjected to GWAS using three different models.

### Data availability

The MAGIC lines and founders are maintained by the sorghum breeding program at Purdue University and are available upon request. Supplemental Material, File S1 contains supplemental figures and tables cited in this article. File S2 contains SNP ID numbers and locations. File S3 contains genotypes for each MAGIC line used for GWAS analysis. File S4 contains genotypes for each Founder line. The GBS raw sequence data are available at the GenBank, and have been assigned accession number PRJNA417037. File S5 contains plant height phenotypic data used for GWAS analysis. File S6 contains full details of the materials and methods employed in this study.

## Results

### Development of MAGIC population

**Figure 1:**
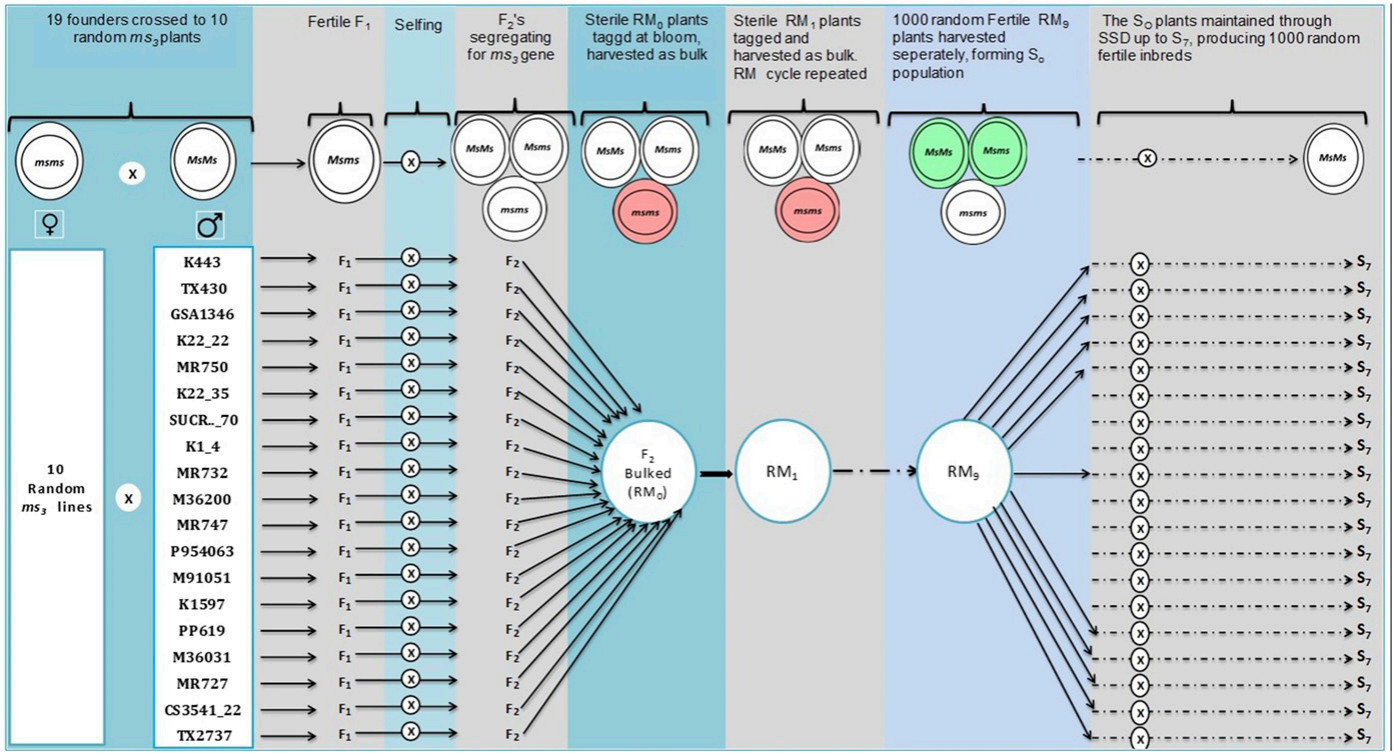
Mating design of the first multi-parent advanced generation intercross (MAGIC) population of sorghum, developed with the aid of genetic male sterility. In this figure, *ms_3_* refers to the male sterile sorghum lines used to introduce sterility in the MAGIC population, RM_0_ to RM_9_ refers to cycles of random mating, S_0_ to S_7_ refers to generations of selfing, and SSD refers to single-seed descent. Initial pairwise hybridization made between 19 founding parents and 10 random sterile plants. Progenies were bulked and random mated for nine cycles, 1000 random lines were generated, and a subset of 200 has been genotyped.

This sorghum MAGIC population was created using 19 diverse founder lines (Table 1) intercrossed randomly through a GMS system, resulting in a population built to suit association studies for complex traits. The GMS in sorghum is conditioned by a nuclear recessive gene that segregates in the population, allowing the creation of random mating populations. Each of the 19 founder lines were crossed to 10 random genetic MS plants in the *ms_3_* source population. The MS source was an open-pollinated population segregating for male sterility, and not a highly inbred genotype. The 190 random MS plants to which the 19 founder lines were crossed (10 × 19) collectively represented the base-population, but unfortunately could not be saved for sequencing. This meant that we could only approximate the estimates of the allelic make-up of the MAGIC population. Each of the resultant F_1_ hybrids were allowed to self-pollinate to generate F_2_ seeds. Equal amounts of F_2_ seeds were pooled from each cross pair and bulked to create the new random mating base population (RM_0_). The RM_0_ population, segregating for the *ms_3_* gene, was planted on an ∼0.25 acre field that first year, and annually for the subsequent 10 seasons, to allow it to intermate randomly. Each year, 1000–1500 random sterile plants in the large intermating population were identified at bloom stage when they could easily be discerned and tagged. Only seeds that set on tagged MS plants, known to be random-mated, were harvested and bulked each generation to form subsequent generations of the population (RM_n_). The sterile plants are easy to identify because they do not shed pollen and have characteristic scaly, whitish or less yellowish, and nonplump anthers (Reddy *et al.* 2005). In the tenth season, random mating was stopped, and the resulting RM_9_ MAGIC population was planted so as to randomly sample 1000 fertile S_0_ plants to start inbreeding. For the next six seasons, each of the 1000 S_0_ plants were self-pollinated and advanced using the SSD method. At the end of the selfing generation, resultant progenies were row-planted in the field so that 1000 S_7_ plant rows were bulked, resulting in 1000 random S_7_ inbred lines. From this set, 200 random S_7_ inbreds were drawn randomly to create a MAGIC subset, used in this study to dissect the structure of the MAGIC population. The mating scheme for the sorghum MAGIC population, described above, is presented in Figure 1. The MAGIC inbreds are currently being maintained by the sorghum breeding program at Purdue University and may be accessed on request.

**Table 1:** Founder lines of the sorghum MAGIC population

Founders	Agronomic Significance
K443	Drought tolerance and early ∼67 d to 50% flowering
TX430	Yield potential, wide adaptation, drought tolerant, and medium ∼71 d to 50% flowering
GSA1346	Drought tolerance and medium ∼85 d to 50% flowering
K22/22	Drought tolerance
MR750	Yield, disease and insect resistance (ergot, midge, and Sugarcane aphid), and general combining ability
K22/35	Drought tolerance
SUCR3680/70	Yield potential and general combining ability
K1/4	Drought tolerance
MR732	Yield potential, medium ∼76 d to 50% flowering, grain quality, and general combining ability
M36200	Yield potential, grain quality, and general combining ability
MR747	Yield potential, grain quality, and general combining ability
P954063	Yield potential, early ∼68 d to 50% flowering, wide adaptation, grain quality, and general combining ability
M91051	Yield potential, grain quality, and general combining ability
K1597	Drought tolerance, early ∼52 d to 50% flowering, general combining ability
PP619	Drought tolerance and yield potential
M36031	Yield potential and grain quality
MR727	Yield potential and grain quality, general combining ability
CS3541-22	Yield potential, medium ∼75 d to 50% flowering, grain mold resistance, grain quality, general combining ability, and wide adaptation
TX2737	Drought tolerance, medium ∼70 d to 50% flowering, and general combining ability

### Genomic features of MAGIC population

**Figure 2:**
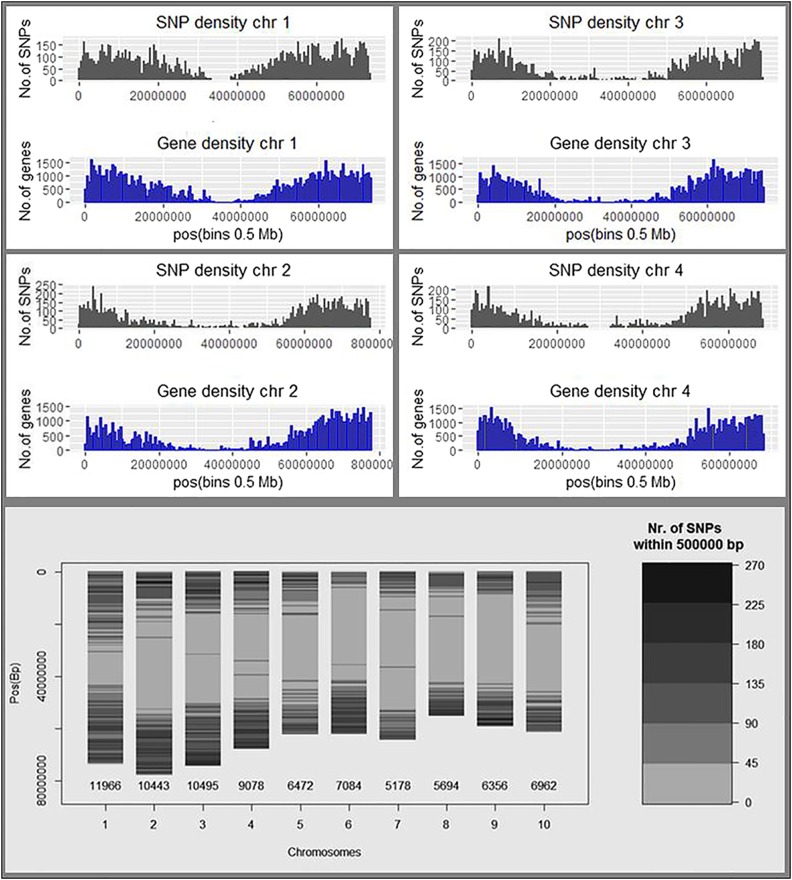
Chromosome-wide distribution of single nucleotide polymorphisms (SNPs) and reference sequence genes. Each of the 10 sorghum chromosomes (chr) is presented with SNP density (in gray) and a corresponding gene density (in blue) plotted below it. Chromosomes 5–10 are shown in Figure S1 in File S1. A summary plot of the number of SNPs per chromosome is presented at the bottom right hand corner of this figure. Pos, position.

Analysis of the GBS data resulted in a total of 79,728 SNPs (excluding those that mapped to super contigs), distributed across the entire sorghum genome and at an average spacing of 9 kb. The distribution of SNPs varied within and among chromosomes. Chromosome 1 showed the highest density of SNPs (11,966 SNPs), with chromosome 7 being the least (5178 SNPS) dense (Figure 2 and Figure S1 in File S1). Chromosome-wide SNPs and gene density analyses revealed that the MAGIC SNPs were higher in regions with high gene density and vice versa, indicating the richness of SNPs in the MAGIC population and their potential usefulness in tagging genes of interest (Figure 2 and Figure S1 in File S1).

**Figure 3:**
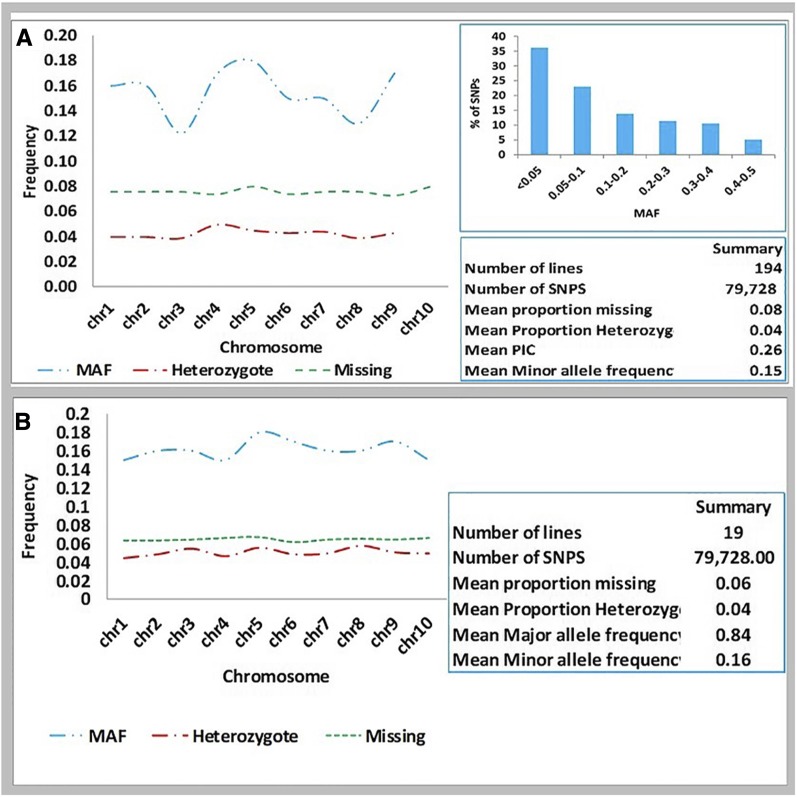
Pattern of genetic features in a subset of sorghum multi-parent advanced generation intercross (MAGIC) population and founder lines. (a) Chromosome-wide distribution of mean minor allele frequency (MAF), mean proportion of heterozygotes, and missing data in the MAGIC subset. The embedded bar chart in (a) depicts the percentage proportion of MAGIC SNPs corresponding to bins or categories of MAF. (b) Chromosome-wide distribution of mean MAF, mean proportion of heterozygotes, and missing data among the 19 founder lines. In (a and b), summary statistics are presented in the embedded text boxes. Nr., number; Pos, position.

The distribution of heterozygotes, MAF, and missing data are presented in Figure 3 and Table S1 in File S1. The average proportions of heterozygotes were 0.039 and 0.044 in the MAGIC subset and founders, respectively (Figure 3, a and b). Mean MAF was 0.15 in the MAGIC subset and 0.16 among the founders, while the mean proportion of missing data were 0.076 and 0.064 in the MAGIC and the founders, respectively (Figure 3, a and b). MAF is useful in assessing the efficiency of the markers in a mapping panel, as shown previously (Pasam *et al.* 2012). Our results showed the mean MAF in the MAGIC panel to vary from 0.12 on chromosome 4 to 0.18 on chromosome 6. About 36% of the MAGIC SNP markers had MAF < 0.05 (Figure 3a), suggesting that a greater percentage (64%) of the SNPs had high MAF and with sufficient polymorphism required for GWAS.

We further estimated the proportion of polymorphic alleles that were shared between the 200 subsets of the MAGIC population and the founders, by first filtering the data to allow > 0.01 MAF with < 10% missing data. A total of 48,327 and 48,545 SNPs were polymorphic among the MAGIC subset and founders, respectively, while 35,507 SNPs segregated in both (Figure S2, a–c in File S1 and Table 2). We expressed the number of shared SNPs relative to the total number of SNPs in the founders to determine the proportion of founder SNPs captured in the MAGIC subset. The proportion of founder alleles segregating within the MAGIC subset varied among chromosomes from a minimum ratio of 0.69 on chromosome 10 to a maximum of 0.79 on chromosome 5, and with a mean of 0.73 (Figure S2, a–c in File S1 and Table 2).

**Table 2:** Chromosome-wide distribution of polymorphic founder alleles that segregated in the MAGIC population

	M_T_	F_T_	M_Only_	F_Only_	M ∩ F	*(M ∩ F)/F_T_
Chr 1	7,279	7,280	1,939	1,940	5,340	0.73
Chr 2	6,510	6,231	1,928	1,649	4,582	0.74
Chr 3	6,315	6,396	1,632	1,713	4,683	0.73
Chr 4	5,379	5,255	1,579	1,455	3,800	0.72
Chr 5	3,718	4,180	770	1,232	2,948	0.71
Chr 6	4,517	4,506	1,066	1,055	3,451	0.77
Chr 7	3,070	3,129	813	872	2,257	0.72
Chr 8	3,375	3,689	822	1,136	2,553	0.69
Chr 9	3,993	3,942	991	940	3,002	0.76
Chr 10	4,171	3,937	1,280	1,046	2,891	0.73
Summary	48,327	48,545	12,820	13,038	35,507	0.73

M_T_ and F_T_ refer to the respective total number of multi-parent advanced generation intercross (MAGIC) and founder alleles that were polymorphic at minor allele frequency > 0.01; M_Only_ and F_Only_ are polymorphic alleles that were unique to the MAGIC and founders, respectively; and M ∩ F are the alleles that are shared between the MAGIC and the founders. * indicates the ratio of shared alleles to total founder alleles, depicting the proportion of founder alleles that segregated in the MAGIC population. Chr, chromosome.

### MAGIC population structure

**Figure 4:**
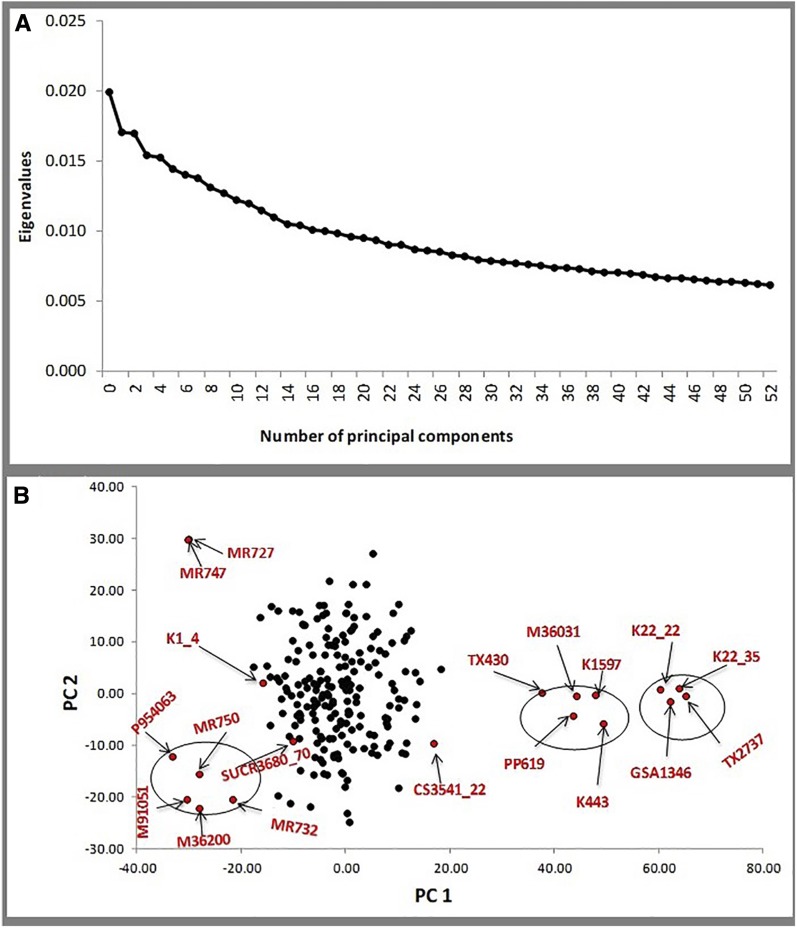
PC analysis of the sorghum MAGIC population. (a) Scree plot of the PCs (*x*-axis) and their contribution to variance (the eigenvalues) *y*-axis). (b) Axes plot of the first two PCs for the MAGIC subset (black dots), including the founders (red dots labeled with founder names). The MAGIC subset appears to form a single group surrounded by three major clusters of founders (black circles around groups of red dots). chr, chromosome; MAGIC, multi-parent advanced generation intercross; PC, principal component.

Population stratification was assessed using multiple statistical approaches including PCA, neighbor-joining tree, and STRUCTURE analyses. The PCA results are presented in Figure 4. The Scree plot showed ∼14 principal components (PCs) before beginning to flatten (Figure 4a), with these first 14 PCs together explaining ∼21% of total variation in the population. That many PCs were required to account for only 21% of total variation in the population indicated that each of the individual PCs explained small, and about the same amount of, variation, suggesting an absence of unique groupings within the population. Because the PC values were about the same, we clustered the population using the first two PCs, which together explained 5% of the total variation. PCA did not show any grouping pattern among the 200 individuals in the MAGIC subset, yet the founders were clustered into three major groups (Figure 4b). Neighbor-joining tree analysis revealed a similar pattern to that observed from PCA (Figure S3, a and b in File S1). When we compared the MAGIC structure to that of the sorghum association panel (SAP), which has been reported in a previous GWAS (Morris *et al.* 2013), as an example of a structured population (Figure S3, c and d in File S1), the difference was remarkably high, with the SAP displaying five distinctive clusters (Figure S3d in File S1), in clear contrast to the MAGIC showing a more uniform distribution of individuals in the population. Further attempts to infer clusters in the MAGIC subset using STUCTURE software (Pritchard *et al.* 2000) yielded no evidence of population structure. A plot of mean posterior probability distribution [lnP(D)] for each run against the number of inferred clusters showed lnP(D) to decrease with an increase in K (Figure 6a, Figure S4a, and Table S2 in File S1), an outcome exactly opposite to that often seen in structured populations. The highest values of lnP(D) were between K = 1 and K = 4, and fluctuated thereafter while descending (Figure S4a and Table S2 in File S1). If there was true population stratification, we would expect small lnP(D) for lower values of K, and this would increase steadily and plateau for larger Ks (Pritchard *et al.* 2000). The bar plot graphics of STRUCTURE revealed no complete assignment of any individual in the MAGIC subset to a specific group (Figure S4b in File S1). Overall, PCA, neighbor-joining tree, and STRUCTURE analyses generated consistent evidence for no subgrouping in the sorghum MAGIC population.

### LD analysis

**Figure 5:**
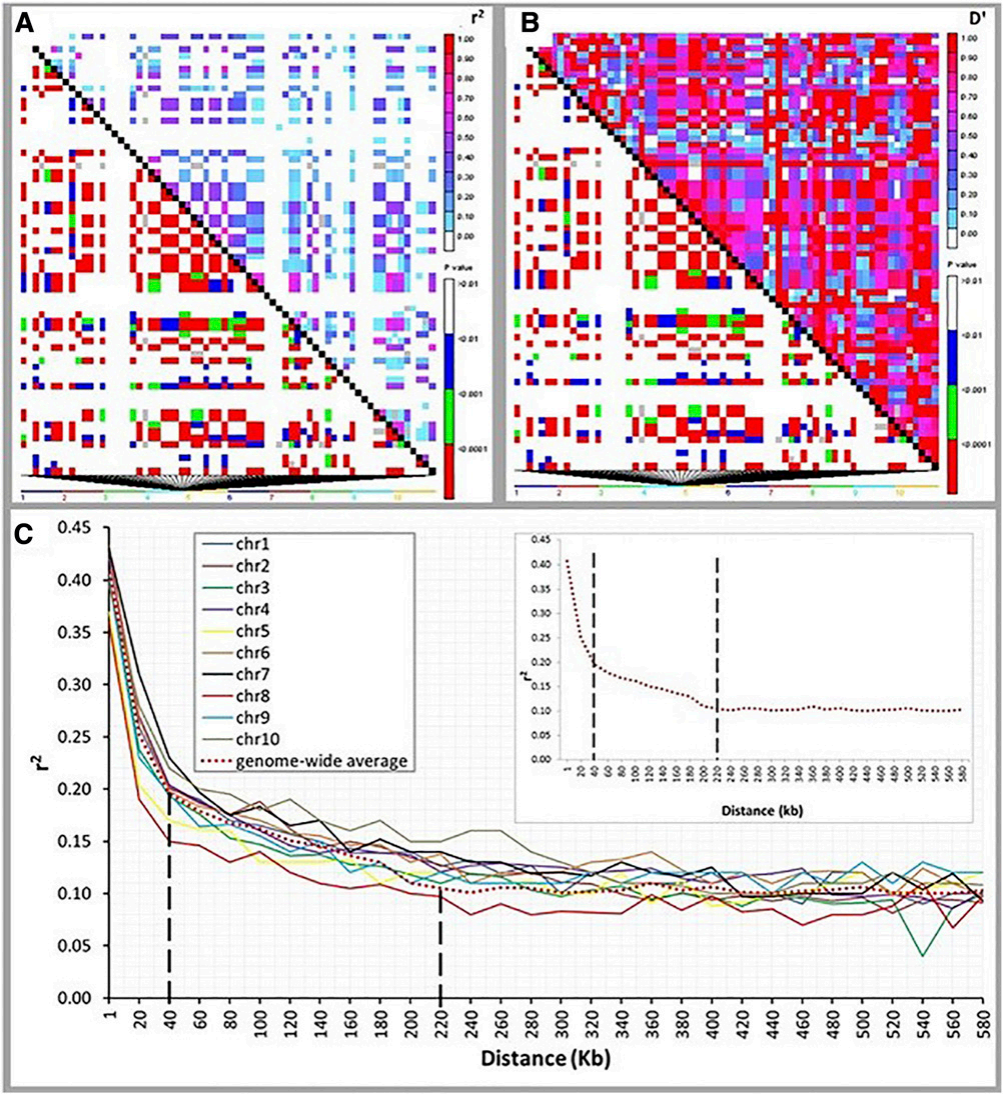
LD patterns. (a and b) LD heatmaps of a segment of the MAGIC genome based on (a) *r*^2^ and (b) D’, with *r*^2^ and D’ shown above diagonals while P-values are below diagonals. Each cell represents the comparison of two pairs of marker sites with the color codes shown for the presence of significant LD. (c) LD decay rate for each chromosome. The top right insert depicts the mean genome-wide decay rate. The vertical dashed lines intersect the decay curve at *r*^2^ = 0.2 and *r*^2^ = 0.1, corresponding to the distances of 40 and 220 kb, respectively, the LD values below which the pairwise linkage between loci dissipates. chr, chromosome; LD, linkage disequilibrium; MAF, minor allele frequency; MAGIC, multi-parent advanced generation intercross; SNP, single nucleotide polymorphism.

LD analysis was conducted to explore the genome landscape of the MAGIC population for association studies. The pattern of LD across the entire genome revealed several haplotype blocks harboring SNPs that are in strong LD (Figure 5, a and b). These blocks are surrounded by regions that have undergone recombination and are therefore not in LD. The P-values associated with the *r*^2^ or D´ (measures of LD), clearly showed the patterns of block fragmentation that are spread across the genome (Figure 5, a and b). Such patterns offer great opportunity for fine mapping, since the SNPs that persistently remain in strong LD despite the opportunity for recombination tend to be tightly associated with the genes of interest.

To estimate the size of the LD blocks or the LD decay rate in the MAGIC population, we plotted the *r*^2^ values against physical distance (kb) for each chromosome (Figure 5c and Table S3 in File S1). We set the LD decay rate at *r*^2^ threshold values of 0.2 and 0.1. These plots provided estimates of LD decay to be at 40 kb when *r*^2^ ≤ 0.2 and 220 kb when *r*^2^ ≤ 0.1, close to the values reported for outcrossing species or highly diverse populations (Morris *et al.* 2013). This implied that that the genomes of MAGIC-derived lines are highly recombined, and therefore well suited for genome-wide gene mapping.

### Mapping plant height genes in sorghum

To verify the suitability of our new sorghum MAGIC population for gene mapping, as predicted from our genetic analysis, we conducted genome-wide association analysis for plant height. This phenotype is a well-studied trait in sorghum, and four major dwarfing genes controlling this trait have been previously described (Brown *et al.* 2008; Morris *et al.* 2013; Thurber *et al.* 2013; Higgins *et al.* 2014). Phenotypic dissection and depiction of genetic variability from a 2 yr plant height data set is summarized in Figure S5 in File S1 and Table 3. Genotypic differences, year-to-year variation and the interaction between genotype and year were highly significant (P ≤ 0.001). The genotypic variance component accounted for most of the phenotypic variation, that is, 75% (2013), 75% (2014), and 65% (2013 and 2014 combined). Repeatability for plant height was ∼85%, which reflected that the trait measurements were fairly consistent between replications as well as across years, an indication of high predictability and, hence, high heritability.

**Table 3:** ANOVA depicting magnitude of genetic variation for plant height among the 200 subset of sorghum MAGIC population

Mean Squares							Variance Components and Repeatability			
	2013		2014		Combined		Source	2013	2014	Combined
Source of var.	d.f.	MS	d.f.	MS	d.f.	MS				
Genotype (G)	199	760***	199	1358***	199	1849***	σ^2^_G_	325	582	395
Replication	1	75	1	812*	1	690*	σ^2^_Y_	—	—	12
Year (Y)	—	—	—	—	1	5187***	σ^2^_G × Y_	—	—	58
G × Y	—	—	—	—	199	269***	σ^2^_e_	110	193	152
Residuals	199	110	199	194	399	152	σ^2^_T_	435	776	618
Total	399		399		799		R	0.86	0.86	0.85
Mean	113		110		183					
Min	113		110		111		Proportion of Components (%)			
Max	230		250		235		Source	2013	2014	Combined
*^a^*SEM_g_	7.4		9.8		6.2		σ^2^_G_	75	75	64
*^a^*SEM_y_	—		—		0.6		σ^2^_Y_	—	—	2
*^a^*SEM_g × y_	—		—		8.7		σ^2^_G × Y_	—	—	9
*^b^*LSD_g_	20.7		27.5		17.1		σ^2^_e_	25	25	25
*^b^*LSD_y_	—		—		1.7		σ^2^_T_	100	100	100
*^b^*LSDg _× y_	—		—		24.2					
CV(%)	9.3		12.7		6.7					

Analysis results are presented for year 2014 and 2014 independently and in combination. *, **, and *** indicate significance at 0.05, 0.01, and 0.001 probability levels, respectively. σ^2^_G_, σ^2^_Y_, σ^2^_G × Y,_ σ^2^_e_, and σ^2^_T_ are the respective variance components for genotype, year, genotype by year interaction, error, and total. “R” is repeatability (analogous to broad sense heritability for estimates in single or few environments) and is a measure of predictability of trait measurements over time. MS, mean squares; d.f., degrees of freedom; Min, minimum; Max, maximum; LSD, least significant difference; CV, coefficient of variation.

a The respective SEM for genotypes, year and the interaction between genotype and year.

b The least significant difference for genotypes, year and the interaction between genotype and year respectively.

**Figure 6:**
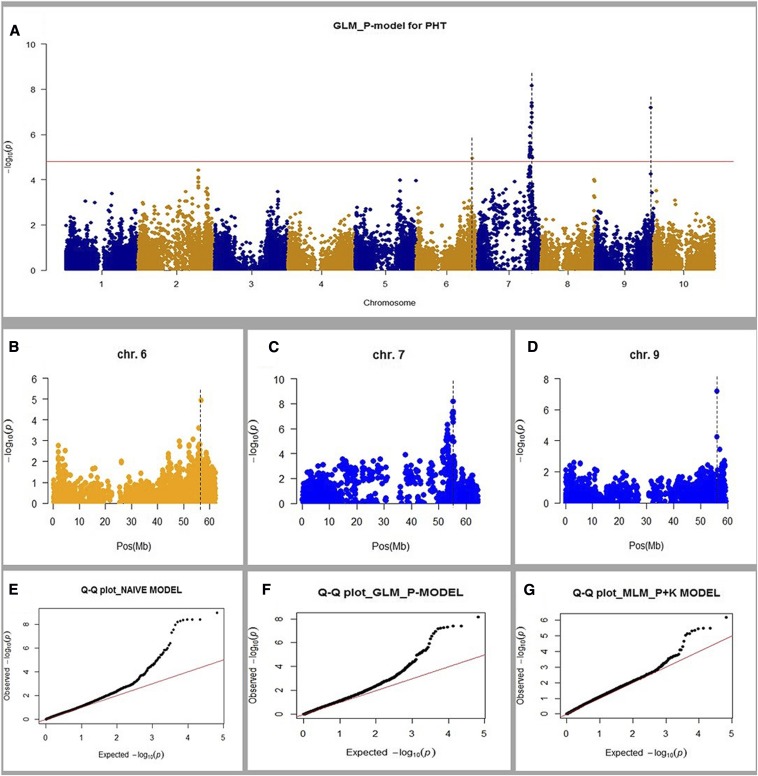
Genome-wide scan for plant height loci. (a) Manhattan plot based on Q-model shows a strong association signal in the plant height gene regions. (b–d) Highlight of peaks for the two known (*DWARF1 and DWARF3*) and one new (*QTL-6*) dwarfing genes: (b) *QTL-6*, (c) *DWARF3*, and (d) *DWARF1*. The vertical dashed lines show the physical positions of the height genes. (e–g) Q-Q plots for the three models: (e) naïve model, (f) Q-model, and (g) Q+K-model. Significant threshold (horizontal line) in (a) was determined based on the average linkage disequilibrium (LD) decay extent in the multi-parent advanced generation intercross (MAGIC) population as: Number of independent tests = reference genome size (730 Mb)/MAGIC LD extent (220 kb); the threshold is then obtained as [0.05/(730 Mb/0.22 Mb)] = 1.50685 × 10^−5^, equivalent to −log10(P) of 4.8.

Genome-wide association analysis based on the GLM (Q model) revealed three significant [−log 10(P) ≥ 4.8] association signals for plant height on chromosomes 6, 7, and 9 (Figure 6a). These signals spanned genomic regions that harbor two known plant height genes, *DWARF1* on chromosome 9 and *DWARF3* on chromosome 7 (Feltus *et al.* 2006; Brown *et al.* 2008; Upadhyaya *et al.* 2012; Morris *et al.* 2013; Thurber *et al.* 2013; Higgins *et al.* 2014), and a potentially new plant height locus (*QTL-6*) on chromosome 6 (Figure 6, b–d). Remapping of these plant height genes signified the potential utility of the sorghum MAGIC panel in gene discovery. Examination of the Q-Q plots from the three GWAS models (GLM-Naïve, GLM-Q, and MLM-Q+K), used in this study to evaluate the effect of these models on gene mapping in the MAGIC population, showed that the Q-Q plot for the Naïve and Q models were very similar, with no serious departure from expectation (Figure 6, e and f), with power to detect the three plant height genes. On the other hand, the Q+K model [mixed linear model (MLM)] showed a better fit than the other two models (Figure 6g), although the signal for the *QTL-6* locus on chromosome 6 could not reach the genome-wide significance threshold (Figure S6 in File S1).

## Discussion

In crops, the potential for high-resolution mapping has not been fully exploited, partly because little progress has been made in designing mapping resources to match the rapid advances in genotyping technologies. Early development of novel genetic resource panels in maize (Dell’Acqua *et al.* 2015; Holland 2015) and rice (Bandillo *et al.* 2013) has helped to initiate many genomic studies that have led to the discovery of genes and gene functions in these crops. In sorghum, QTL are still being mapped based on traditional biparental mapping populations with limited allelic combinations and recombination, although random mating populations have long been used in recurrent selection programs (Eberhart 1972; Maunder 1972; Reddy and Stenhouse 1994). In the past, the complexity of the genetics in such broad-base populations limited their usage as a gene mapping resource. More recently, the development of high-throughput sequencing technologies, and greater statistical power, have generated new interest and attention in creating unique mapping populations to take advantage of the new genomic tools. MAGIC populations are the latest mapping resources with the potential to overcome some of the limitations of biparental populations.

This MAGIC population, the first described in sorghum, is rich in multiple allelic combinations from its diverse founders, making it a good mapping resource and a breeding pool for cultivar development. The sorghum population derives its uniqueness in the fine structure brought about due to the multiple cycles of random mating, with the aid of GMS in its crossing scheme. GMS in sorghum is conditioned by a single nuclear recessive allele in homozygous condition, designated with a series of alleles as *ms_1_*–*ms_7_* (Rooney and Klein 2000; Reddy *et al.* 2005). Male sterility induced by the *ms_3_* and *ms_7_* alleles are very stable and have been widely used in population improvement (Reddy and Stenhouse 1994; Reddy *et al.* 2005). An advantage of this system is that, once introduced in the population, the *ms* gene facilitates intercrossing readily among founding parents in a population that is planted in isolation, without the need for the maintenance of complex pedigrees. Multiple cycles of random mating can therefore be achieved, thereby speeding up the process a bit more and eliminating tedious manual emasculation and crossing. More importantly, the risk of assortative mating (Templeton 2006; Kole 2013) is significantly reduced, owing to intermating that is facilitated by wind pollination through secondary and tertiary growths (tiller plants) that allow random mating among different maturity groups, compared to previously reported MAGIC populations in other crops that relied on mechanical crossing schemes other than GMS.

The sorghum MAGIC SNP panel consists of 79,000 SNPs spanning the entire genome, most of which are in densely populated gene rich regions, with few around pericentromeric regions (Figure 2). Sorghum chromosomes are known to contain large pericentromeric regions spanning ∼50% of the genome, and these regions are characterized by low gene density and very low rates of recombination (Evans *et al.* 2013). In terms of efficiency, the sorghum MAGIC SNP panel showed variable distribution of MAFs per chromosome, the average MAF being 0.15. In most cases, MAF ≤ 0.05 is considered appropriate for any SNP mapping panel (The International HapMap Consortium 2005). MAF estimation is important in GWAS because the least common alleles in the population are difficult to detect with low statistical power and, if detected, are very prone to false-positive associations (Tabangin *et al.* 2009). It would require impracticably large population sizes to precisely detect associations of minor alleles with traits. For this reason, SNPs with MAF ≤ 0.05 are often excluded from GWAS analysis. For our sorghum MAGIC SNP panel, excluding 36% of SNPs with MAF < 0.05, leaves ∼51 K informative SNPs spanning the genome at an average spacing of 14 kb, which is sufficient and efficient for GWAS. However, mean residual heterozygosity (0.039) in the sorghum MAGIC population was higher than expected for S_7_ inbreds, and not comparable to the values observed in a maize MAGIC population (Dell’Acqua *et al.* 2015). This higher level of heterozygosity may reflect contamination and poor genotyping integrity, suggesting perhaps that this SNP panel should be filtered to reduce the percentage of heterozygous loci. The proportion of founder alleles that segregated in the MAGIC population was 73%, a somewhat lower value compared to that observed in maize (Dell’Acqua *et al.* 2015). This may have been caused by unaccounted alleles from the random plants from the MS base population germplasm to which the founder lines were intercrossed, given that alleles from that source were not tracked, but evenly distributed in the resultant MAGIC population.

The results of this study provided evidence for the absence of a population structure, which is consistent with other MAGIC populations that have been previously described in other crop plants (Cavanagh *et al.* 2008; Huang *et al.* 2012; Bandillo *et al.* 2013; Mackay *et al.* 2014; Dell’Acqua *et al.* 2015). Population structure affects GWAS results as it is the major source of false associations, and it is the reason for the development of complex statistical models like the MLM and efficient mixed-model association (EMMA) algorithms to try to account for the problem (Kang *et al.* 2008; Zhang *et al.* 2010). Unfortunately, these models, while they are useful in reducing the problem, do not completely resolve the basic problem with structured populations. With nonstructured populations, the burden of complex modeling is eliminated and association signals are usually real.

Random mating increases the frequency of detectable recombinants in the population (Beavis *et al.* 1992) and enhances LD decay. Our results showed a generally low LD, the maximum genome-wide average having an *r*^2^ value of 0.4. This LD diminished quickly in our population to 0.2 when two loci were 40 kb apart, and down to 0.1 when the loci were 220 kb apart, a decay rate that is comparable to those observed in natural populations (Morris *et al.* 2013). The faster LD decay achieved in our population through the multiple RM cycles was made possible by random mating through the use of the GMS system. Faster LD decay rate means that the haplotype blocks are smaller due to enhanced recombination, and that the loci that remain in LD at this point are truly linked. This is the origin of the high-resolution mapping commonly implied in association studies.

By remapping some of the previously identified plant height genes—*DWARF1* (chromosome 9), *DWARF2* (chromosome 6), and *DWARF3* (chromosome 7) (Figure 6, b–d)—we show the potential of the sorghum MAGIC population as a powerful mapping resource. An early genetic study found four loci affecting sorghum height: *DWARF1*, *DWARF2*, *DWARF3*, and *DWARF4* (Quinby and Karper 1954). The *DWARF3* gene lying within 58.56–58.57 Mbp on chromosome 7 has been cloned and it encodes an auxin efflux carrier, PGP19 (Multani *et al.* 2003; Higgins *et al.* 2014). We mapped a plant height QTL within a 56–58 Mbp region on chromosome 7 (Figure 6c). Recently, *DWARF1*, located within 55–57 Mbp on chromosome 9, was cloned and found to encode a putative membrane protein of unknown function that is highly conserved in plants (Hilley *et al.* 2016; Yamaguchi *et al.* 2016). The present study mapped one plant height QTL within the same region of 56–57 Mbp (Figure 6d). Additionally, *DWARF2* has been found to be associated with a QTL on chromosome 6, but this QTL has not yet been cloned (Feltus *et al.* 2006; Klein *et al.* 2008; Mace and Jordan 2011; Upadhyaya *et al.* 2012; Morris *et al.* 2013). These studies suggested that *DWARF2* is positioned within 43–45 Mbp on chromosome 6, but our QTL on the same chromosome is positioned within 56–57 Mbp, > 10 Mbp away, indicating the new plant height QTL (*QTL-6*) that we detected on chromosome 6. On the other hand, *DWARF4* has not been genetically mapped conclusively to a linkage group, although some reports indicate a probable location on chromosome 9 (Brown *et al.* 2008).

We further examined the Q-Q plots of three commonly used GWAS models that are based on GLM and MLM algorisms to evaluate spurious associations that could emanate from residual undetected structure in our MAGIC panel. All three models (Naïve, Q-model, and Q+K-model) did not show any extreme deviation from expectations. In GWAS, early departure from the diagonal identity line of a Q-Q plot usually suggests that the assumed distribution is not correct and is a signal for the presence of false associations (Pearson and Manolio 2008; Voorman *et al.* 2011). The Q+K-model showed a generally more reduced statistical significance than the other two models, with the association peak for the *QTL-6* locus on chromosome 6 not reaching a genome-wide statistical threshold.

In summary, this sorghum MAGIC population provides unique opportunities. It has highly dense genome-wide polymorphic alleles spanning gene-rich regions. Recombination, enhanced through random mating, has improved its mapping resolution, showing no evidence of population stratification. Its utility for gene mapping has been demonstrated by remapping two previously identified plant height loci on chromosomes 7 and 9, and a new height QTL on chromosome 6. The results reported suggest that the new sorghum MAGIC population is a useful resource for the further exploitation of traits whose variation is captured in the population. Highly inbred and genetically diverse, the immortal lines that make up the population would serve as potential sources of germplasm for improved sorghum lines and hybrids. The sorghum crop is endowed with rich genetic diversity. With robust adaptive traits and a small genome size of 730 Mbp (∼25% the size of maize or sugarcane) that is fully sequenced (Paterson *et al.* 2009), sorghum is an attractive model for genomic studies of C4 grasses. The crop is related to many cereals including maize, such that new innovations, including dissection of causal variants in its genome, may enhance the use of syntenic knowledge with relative ease in homologous gene discoveries among related species (Hamblin *et al.* 2004; Paterson 2008). With more investment in further genetic studies in the crop, the sorghum genome could be an asset for the wider application of genomic tools toward enhancing crop productivity, as well as tolerance to biotic and abiotic stresses.

## Supplementary Material

Supplemental material is available online at www.g3journal.org/lookup/suppl/doi:10.1534/g3.117.300248/-/DC1.
