# Supplementary material for: Mating Design and Genetic Structure of a Multi-Parent Advanced Generation Intercross (MAGIC) Population of Sorghum (Sorghum bicolor (L.) Moench)
Source: G3 (Bethesda). 2017 Nov 17;8(1):331–41. doi: 10.1534/g3.117.300248 (PMC5765360; doi:10.1534/g3.117.300248)
Supplement: Supplementary file 1 [file 331FileS1.docx]

# Supplementary Tables and Figures

# Mating design and genetic structure of a multi-parent advanced generation inter-cross (MAGIC) population of Sorghum (*Sorghum bicolor* (L.) Moench)

Patrick O. Ongom and Gebisa Ejeta

Correspondence to: gejeta@purdue.edu

**Table S1**

Chromosome-wise distribution of major and minor allele frequencies, level of heterozygosity and proportion of missing data in the MAGIC subset.

| Chromosome | ^a^Maj.AF | ^b^MAF | ^c^Het | ^d^Missing | ^e^PIC |
| --- | --- | --- | --- | --- | --- |
| chr1 | 0.85 | 0.15 | 0.04 | 0.08 | 0.26 |
| chr2 | 0.84 | 0.16 | 0.04 | 0.08 | 0.27 |
| chr3 | 0.84 | 0.16 | 0.04 | 0.08 | 0.27 |
| chr4 | 0.89 | 0.12 | 0.04 | 0.07 | 0.19 |
| chr5 | 0.83 | 0.17 | 0.05 | 0.08 | 0.28 |
| chr6 | 0.82 | 0.18 | 0.05 | 0.07 | 0.30 |
| chr7 | 0.85 | 0.15 | 0.04 | 0.08 | 0.26 |
| chr8 | 0.84 | 0.15 | 0.04 | 0.08 | 0.27 |
| chr9 | 0.87 | 0.13 | 0.04 | 0.07 | 0.23 |
| chr10 | 0.83 | 0.17 | 0.04 | 0.08 | 0.28 |
| Mean | 0.85 | 0.15 | 0.04 | 0.08 | 0.26 |

^a^Major allele frequency; ^b^Minor allele frequency; ^c^Proportion heterozygous; ^d^Proportion missing; ^e^Polymorphic information content

**Table S2**

Posterior distribution (lnP(D)) for STRUCTURE runs with 16 different populations assumed (k), each k replicated five times.

| Inferred groups (K) | ln P(D)1 | ln P(D)2 | ln P(D)3 | ln P(D)4 | ln P(D)5 | Mean ln P(D) |
| --- | --- | --- | --- | --- | --- | --- |
| k1 | -4225106 | -4223848 | -4223992 | -4224749 | -4224460 | -4224431 |
| k2 | -4212007 | -4194773 | -4187602 | -4187542 | -4191313 | -4194647 |
| k3 | -4148457 | -4254648 | -4163845 | -4395474 | -4150542 | -4222593 |
| k4 | -4712610 | -8721054 | -5414095 | -5345617 | -4190539 | -5676783 |
| k5 | -4136380 | -22400414 | -4249810 | -5168203 | -5574090 | -8305779 |
| k6 | -73053323 | -20722306 | -5336731 | -5378799 | -4164363 | -21731105 |
| k7 | -23766379 | -16862217 | -9402188 | -12906575 | -7471352 | -14081742 |
| k8 | -6560297 | -90558587 | -88774631 | -60972370 | -199067885 | -89186754 |
| k9 | -127216343 | -210538912 | -107557793 | -21018077 | -119209446 | -117108114 |
| k10 | -99851731 | -124501994 | -44113023 | -64767741 | -106028151 | -87852528 |
| k11 | -114090930 | -171137186 | -30612641 | -44286520 | -202947558 | -112614967 |
| k12 | -965262204 | -122807132 | -558914242 | -129092143 | -428842945 | -440983733 |
| k13 | -201756311 | -40668605 | -462322950 | -463559068 | -417525944 | -317166576 |
| k14 | -547297548 | -1054524170 | -185555143 | -474112680 | -710060119 | -594309932 |
| k15 | -1841006902 | -354824765 | -332001985 | -1186159124 | -94469854 | -761692526 |
| k16 | -308046599 | -704397968 | -311047002 | -270847565 | -291191459 | -377106119 |

ln P(D) is a posterior probability distribution showing the likelihood that the population is subdivided into any of the K values assumed (k=1 to k=16)

**Table S3**

Rate of linkage disequilibrium decay at 20 Kb interval for each chromosome.

| Distance (Kb) | chr1 | chr2 | chr3 | chr4 | chr5 | chr6 | chr7 | chr8 | chr9 | chr10 | Mean |
| --- | --- | --- | --- | --- | --- | --- | --- | --- | --- | --- | --- |
| 1 | 0.43 | 0.43 | 0.41 | 0.42 | 0.37 | 0.43 | 0.43 | 0.36 | 0.40 | 0.40 | 0.41 |
| 20 | 0.26 | 0.27 | 0.24 | 0.27 | 0.20 | 0.27 | 0.31 | 0.19 | 0.23 | 0.28 | 0.25 |
| 40 | 0.20 | 0.20 | 0.19 | 0.20 | 0.17 | 0.20 | 0.23 | 0.15 | 0.20 | 0.22 | 0.20 |
| 60 | 0.19 | 0.19 | 0.18 | 0.19 | 0.16 | 0.18 | 0.20 | 0.15 | 0.16 | 0.20 | 0.18 |
| 80 | 0.17 | 0.18 | 0.15 | 0.18 | 0.16 | 0.18 | 0.18 | 0.13 | 0.17 | 0.20 | 0.17 |
| 100 | 0.17 | 0.19 | 0.15 | 0.16 | 0.13 | 0.17 | 0.18 | 0.14 | 0.16 | 0.18 | 0.16 |
| 120 | 0.16 | 0.16 | 0.14 | 0.15 | 0.13 | 0.16 | 0.17 | 0.12 | 0.14 | 0.19 | 0.15 |
| 140 | 0.15 | 0.14 | 0.14 | 0.14 | 0.13 | 0.16 | 0.17 | 0.11 | 0.15 | 0.17 | 0.15 |
| 160 | 0.14 | 0.15 | 0.13 | 0.14 | 0.13 | 0.15 | 0.14 | 0.11 | 0.12 | 0.16 | 0.14 |
| 180 | 0.14 | 0.14 | 0.13 | 0.14 | 0.11 | 0.15 | 0.15 | 0.11 | 0.13 | 0.17 | 0.13 |
| 200 | 0.14 | 0.13 | 0.12 | 0.14 | 0.12 | 0.13 | 0.14 | 0.10 | 0.11 | 0.15 | 0.11 |
| 220 | 0.13 | 0.13 | 0.11 | 0.12 | 0.12 | 0.14 | 0.14 | 0.10 | 0.12 | 0.15 | 0.11 |
| 240 | 0.13 | 0.12 | 0.12 | 0.13 | 0.11 | 0.11 | 0.13 | 0.08 | 0.11 | 0.16 | 0.10 |
| 260 | 0.12 | 0.12 | 0.12 | 0.13 | 0.11 | 0.12 | 0.13 | 0.09 | 0.11 | 0.16 | 0.11 |
| 280 | 0.13 | 0.12 | 0.10 | 0.13 | 0.11 | 0.12 | 0.12 | 0.08 | 0.11 | 0.14 | 0.11 |
| 300 | 0.10 | 0.11 | 0.10 | 0.13 | 0.10 | 0.12 | 0.12 | 0.08 | 0.11 | 0.13 | 0.10 |
| 320 | 0.12 | 0.11 | 0.10 | 0.12 | 0.10 | 0.13 | 0.12 | 0.08 | 0.12 | 0.12 | 0.10 |
| 340 | 0.11 | 0.10 | 0.11 | 0.12 | 0.12 | 0.13 | 0.13 | 0.08 | 0.11 | 0.11 | 0.10 |
| 360 | 0.11 | 0.09 | 0.10 | 0.12 | 0.09 | 0.14 | 0.12 | 0.10 | 0.12 | 0.11 | 0.11 |
| 380 | 0.11 | 0.10 | 0.10 | 0.12 | 0.11 | 0.12 | 0.12 | 0.08 | 0.12 | 0.11 | 0.10 |
| 400 | 0.12 | 0.09 | 0.10 | 0.11 | 0.09 | 0.11 | 0.13 | 0.10 | 0.12 | 0.10 | 0.11 |
| 420 | 0.10 | 0.10 | 0.09 | 0.12 | 0.09 | 0.12 | 0.10 | 0.08 | 0.12 | 0.10 | 0.10 |
| 440 | 0.10 | 0.09 | 0.10 | 0.12 | 0.10 | 0.11 | 0.10 | 0.09 | 0.10 | 0.10 | 0.10 |
| 460 | 0.09 | 0.10 | 0.10 | 0.12 | 0.10 | 0.12 | 0.10 | 0.07 | 0.12 | 0.11 | 0.10 |
| 480 | 0.12 | 0.09 | 0.09 | 0.10 | 0.11 | 0.12 | 0.10 | 0.08 | 0.11 | 0.11 | 0.10 |
| 500 | 0.12 | 0.10 | 0.09 | 0.10 | 0.12 | 0.12 | 0.10 | 0.08 | 0.13 | 0.11 | 0.11 |
| 520 | 0.10 | 0.08 | 0.09 | 0.10 | 0.10 | 0.10 | 0.12 | 0.09 | 0.11 | 0.12 | 0.10 |
| 540 | 0.09 | 0.09 | 0.04 | 0.10 | 0.10 | 0.12 | 0.10 | 0.11 | 0.13 | 0.11 | 0.10 |
| 560 | 0.10 | 0.09 | 0.09 | 0.09 | 0.11 | 0.11 | 0.12 | 0.07 | 0.12 | 0.11 | 0.10 |
| 580 | 0.10 | 0.09 | 0.10 | 0.10 | 0.12 | 0.10 | 0.09 | 0.10 | 0.12 | 0.11 | 0.10 |

**(b)**

**(a)**

**Figure S1**

Genome-wide distribution of NPs. (a) Number and percent distributions of SNPs shown on the left and right panel respectively. (b) Chromosomes 5-10, showing SNP density (in gray) and a corresponding gene density (in blue) plotted below it

**Figure S2**

Distribution of founder SNP alleles that were captured in the subset of MAGIC population. (a) Chromosome-wise variation in number of founder SNPs, MAGIC SNPs and the SNPs shared between both (b) Percentage distribution of shared alleles per chromosome, with the horizontal dash lines indicating the average proportion (0.73) of founder alleles captured in the MAGIC subset (c) Venn diagram summarizing the overall proportion of polymorphic MAGIC SNPs, polymorphic founder SNPs and SNPs that were shared between both. In panel (c), M_Only_ and F_Only_ refer to polymorphic alleles that were unique to the MAGIC and founders respectively.


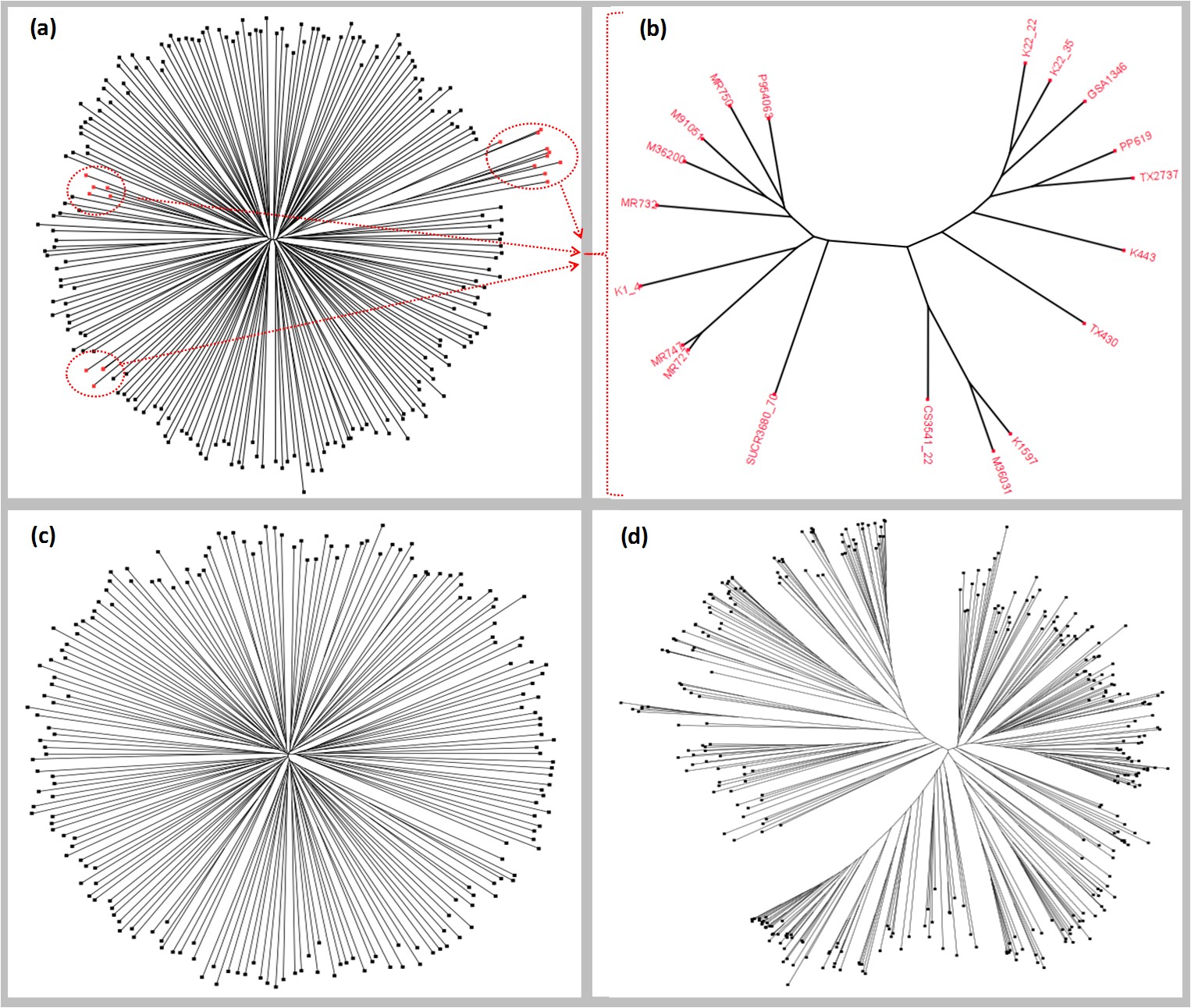


**Figure S3**
Population clustering by Neighbor-Joining tree. (a) MAGIC population representatives (dark dots) including founders (red dots). (b) The 19 founding parents clustered separately, depicting the inherent diversity; (c) MAGIC population excluding founders, the structure is here compared with the sorghum association panel (SAP, d) as an example of a structured population. The SAP cluster was drawn using SNPs data provided in (Morris *et al.* 2013).


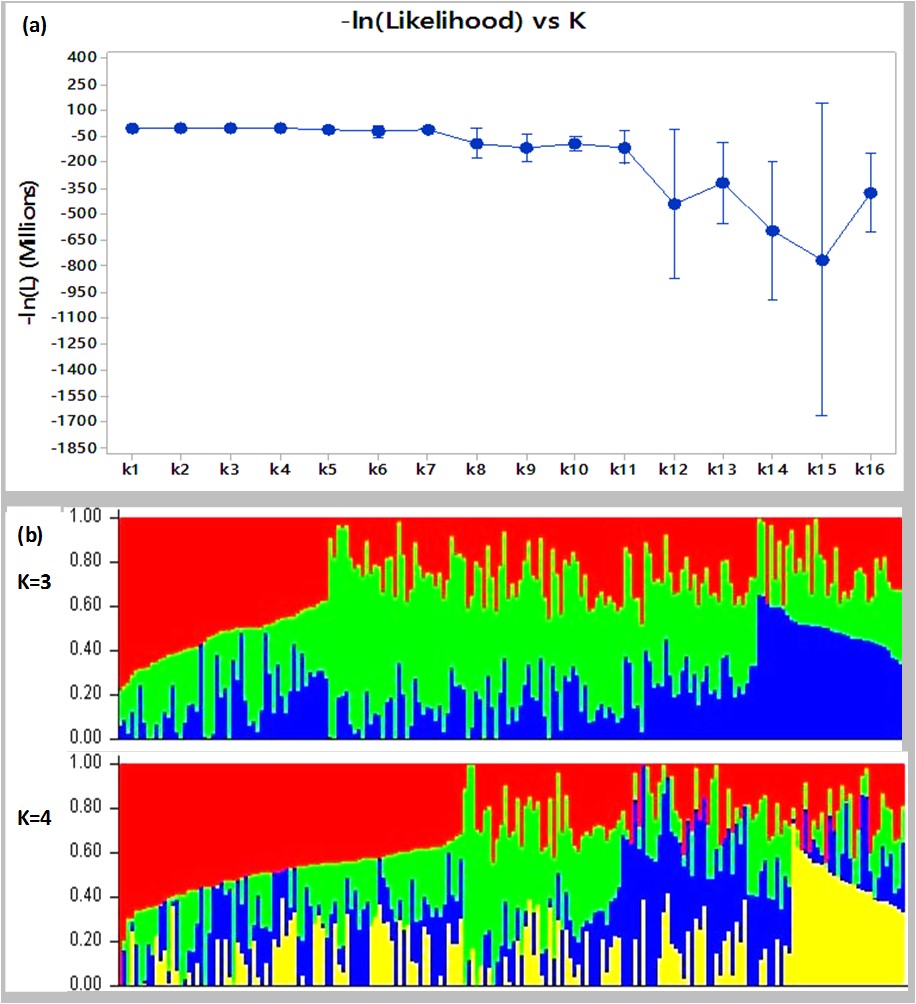


**Figure S4**
Genetic structure of the MAGIC population analysis. (a) Posterior probability, lnP(D), as a function of the number of subpopulations (K). (b) Population structure for K = 3 and K=4. Each vertical line in (b) represents one accession, and the color composition displays the probability of belonging to each subpopulation defined by STRUCTURE.

**Figure S5**

Phenotypic distribution for plant height (a) Histogram of plant height (PHT) (b) Box plot for the distribution of plant height by year.

**QTL-6**

**QTL-6**

**QTL-6**

**Figure S6**

Association signals for three plant height genes based on three regression models (a) GLM-Naïve (model does not account for population structure) (b) GLM-P (general linear model with population structure accounted for using principal components) (c) MLM-PK (Mixed linear model with both structure and relatedness accounted for using PC and kinship matrices). *QTL-6* is potentially new plant height gene located 10MBb away from *DWARF2.*
