# Supplementary material for: Mating Design and Genetic Structure of a Multi-Parent Advanced Generation Intercross (MAGIC) Population of Sorghum (Sorghum bicolor (L.) Moench)
Source: G3 (Bethesda). 2017 Nov 17;8(1):331–41. doi: 10.1534/g3.117.300248 (PMC5765360; doi:10.1534/g3.117.300248)
Supplement: Supplementary file 6 [file 331FileS6.docx]

## MATERIALS AND METHODS:

### Plant Materials

The MAGIC population was constructed from nineteen founder sorghum lines with broad range of useful agronomic traits including early maturity, broad adaptation, yield potential, drought tolerance, pest and disease resistance, and grain quality. The founder lines represent a diverse set of sorghum genotypes derived from genetic resource centers as well as from different geographical regions (**Table 1**). Seeds of founder lines are maintained by Purdue university sorghum research program. The nineteen founders were initially inter-mated and recombined with the aid of a genetic male sterility system that facilitated random mating for several cycles before deriving resultant one thousand random inbreds through a single seed descent (SSD) method of inbreeding. A subset of two hundred S_7_ inbred lines were pooled from the MAGIC population for use in this study.

**Plant height phenotyping**

The two hundred S_7_ inbreds were phenotyped for plant height at the Agronomy Center for Research and Education (ACRE), Purdue University during the summer of 2013 and 2014. The experiment was designed as a randomized complete block design (RCBD) with two replications in each of the two years. Plant height was measured at full maturity on a plot-by-plot basis and mean plant height was averaged across replications and years.

### Genotyping and data processing

Seeds from the 200 subset of MAGIC population and founder lines were grown in plastic trays (40cm x 20cm) filled with vermiculate soil (Therm-O-Rock) in a greenhouse. Two hundred grams of fresh plant tissue per genotype were sampled from two-week-old seedlings. The tissue was lyophilized and pulverized with the aid of metal balls using a MM 301 TissueLyser (Retsch^®^). DNA was then extracted using DNeasy miniprep kits (QIAGEN) using the manufacturer’s protocol. DNA quality of each sample was checked using both NanoDrop 1000 (Thermo scientific) and gel electrophoresis. Thirty µl aliquots of DNA at concentration of 50-100 ng/µl in a 96-well plate were sent to the genomic facility at Cornell University for genotyping using a high throughput genotyping-by-sequencing (GBS) platform (Elshire *et al.* 2011). In this protocol, GBS libraries are constructed by reducing genome complexity using ApeKI restriction enzyme. Sequencing on an Illumina HiSeq, generated 720,142,633 good barcoded reads, resulting in 4,122,598 tags after merging multiple reads and allowing only tags with a total count greater than or equal to three (the minimum number of times a tag must be present to be considered as output). Individual genotypes with less than 10% of the mean reads coming from the lane on which they were sequenced were regarded as failed samples and thus excluded. Out of the 219 samples (200 MAGIC subset + 19 founders), six samples (3%) failed, leaving 213 (194 MAGIC subset + 19 founders).

Tags from successful samples were aligned to the sorghum reference genome, ‘*Sorghum bicolor* v2.1’ (Paterson *et al.* 2009). The reference genome was indexed and alignment generated with the software package BWA Version: 0.7.8-r455 (Li and Durbin 2009). Of the 4,122,598 tags used, 2,763,236 (67.0%) were aligned to unique positions, while 457,904 (11.1%) were aligned to multiple positions and 901,458 (21.9%) could not be aligned. Using the GBS analysis pipeline (Bates *et al.* 2015), tags aligned to the same physical position on the reference genome were compared against each other, and SNPs from each alignment were called and stored in a HapMap file format. During SNP calling, the following criteria were used: the average sequencing error rate was set at 0.01 (used to decide between heterozygous and homozygous calls); Minimum locus coverage was set at 0.1 (the proportion of genotypes with at least one tag at the locus); maximum number of SNPs per chromosome was set at 2,000,000; minimum minor allele frequency was set at $\geq$ 0.01; threshold mismatch rate above which the duplicate SNPs won't be merged was set to default, 0.05. The MAGIC SNP data can be obtained from (the link will be provided later).

### SNP distribution and diversity analysis

To gauge genomic coverage of the discovered SNPs, we generated chromosome-wise density plots for SNPs and the reference sequence genes using ggplot2 (Wickham 2009) and synbreed (Wimmer *et al.* 2012) R-packages. Gene positions used in density plots were extracted from the reference sequence (Sbicolor v2.1), by utilizing the “Download Truck Data” option of the sorghum genome browser found at <http://plant.psc.riken.jp/cgi-bin/gb2/gbrowse/Sbicolor/?name=Chr01>. Further assessment of SNP polymorphism, proportion of heterozygotes, minor allele frequency (MAF) and missingness was conducted in TASSEL: version 5.0 (Bradbury *et al.* 2007), and line graphs summarizing genome-wide distribution of these parameters were generated. To determine the proportion of founder alleles that were captured in the MAGIC subset, the MAGIC and founder SNPs were filtered separately, allowing minor allele frequency (MAF) > 0.01 and 10% missing data and the number of polymorphic SNPs in both panels for each chromosome were counted. Using conditional formatting tools and Visual Basic for Applications (VBA) in Excel Macros, polymorphic SNPs that were shared between the MAGIC subset and parents were counted. A Venn diagram was then used to summarize the relationships. The number of shared SNPs was expressed relative to the number of SNPS in the founder set, to obtain the proportion of founder alleles that are captured in the MAGIC population.

### MAGIC population structure analysis

To assess the pattern of genetic structure of the MAGIC population, principal component analysis (PCA), neighbor joining tree and structure analyses were conducted. PCA scores were generated in TASSEL and exported to Microsoft Excel where visualization graphics were produced. The number of principal components (PCs) capturing the most variation in the population was determined using a scree plot, utilizing PCs and Eigen values generated from TASSEL. To further examine the structure of the population and generate supportive evidences, a cladogram (a phylogenetic analysis tool in TASSEL) was used to cluster the MAGIC subset and that of the founders. The cladogram was generated based on a neighbor-joining algorithm. To support our hypothesis that the random mating cycles incorporated in the development process of the MAGIC population would reduce population stratification, we compared the MAGIC population structure to that of the sorghum association panel (SAP), as an example of a structured population. We obtained SAP SNPs data provided in (Morris *et al.* 2013) and we generated the population cluster in TASSEL for this comparison. Finally, we verified the consistency of results from PCA and Tree analyses, by conducting structure analysis using STRUCTURE software (Pritchard *et al.* 2000). We ran STRUCTURE with the admixture model and a burn-in period of 5000 and 5000 Markov Chain Monte Carlo (MCMC) repetitions. A search for a probable number of groupings (K) in the population was determined by first running 16 independent inferred K values (K=1 to 16), with five replications per K value, and plotting the mean posterior probability distribution (lnP(D)) for each run against the number of inferred clusters. STRUCTURE bar charts for different assumed K values were generated to visualize any possibility of population structure in the MAGIC population.

### Linkage disequilibrium analysis

SNPS were first filtered in Tassel, allowing 10% missing data and minimum minor allele frequency of 0.05. A Linkage disequilibrium (LD) heat map was generated for the entire genome, with heterozygous calls ignored and a default sliding window of 50 used. LD decay rate was evaluated on a chromosome-by-chromosome basis. A measure of LD (r^2^) and pairwise distance between SNPs were generated in TASSEL and exported to “R version 3.0.3” (R Core Team 2014), where scripts were written to generate LD decay plots for each chromosome. Mean LD per chromosome was calculated after every 20 Kb interval, and the average genome-wide decay rate estimated by averaging LD in each interval across all chromosomes. A line graph was used to clearly display overlay of chromosome-specific and mean genome-wide LD decay rates.

### GWAS for plant height

To evaluate the effectiveness of the sorghum MAGIC population for use in gene mapping, we collected plant height data over two years from a subset of the MAGIC population and performed GWAS. Three different models were performed in TASSEL v.5.2.23 to find which one best fit our plant height data set with minimal spurious association based on the general formula:

$y = \mu+X\beta+M\alpha+ Zk + e$

where y is a response vector for phenotypic values, $\mu$ is the total mean, *β* is a vector of fixed effects regarding population structure estimated using principal components (PCs), $\alpha$ is the vector of fixed effect for markers, $k$ is the vector of random effects for kinship and $e$ is the vector of residuals, while X, M and Z are the incidence matrices relating individuals to β, $\alpha$ and $k$, respectively. Individual terms in the general formula above were excluded accordingly when fitting the three models: (i) Naive model: General linear model (GLM) without accounting for both structure and kinship; (ii) Q-model: GLM with PCs as correction for population structure; (iii) Q+K-model: Mixed linear model (MLM) with PCs and kinship (K)-matrix as correction for population structure. Model statistics from TASSEL were exported to R statistical package (R Core Team 2014) where scripts were written to generate Manhattan and quantile-quantile (QQ) plots.

A genome-wide significant threshold was established according to Matthies *et al.* (2014) and Zhang *et al.* (2015), by utilizing on the average LD decay extent in the MAGIC population as follows: Number of independent tests = Reference genome size (730Mb)/MAGIC LD extent (220kb). Using 0.05 as the desired probability of type I error, the threshold was then obtained as [0.05/((730Mb/0.22Mb))] = 1.50685 x 10-5, equivalent to –log10(P) of 4.8.

**Data availability statement**

The MAGIC lines and founders are maintained by the sorghum breeding program at Purdue University and are available upon request. File S1 contains supplemental figures and tables cited in this article. File S2 contains SNP ID numbers and locations. File S3 contains genotypes for each MAGIC line used for GWAS analysis. File S4 contains genotypes for each Founder line. The GBS raw sequence data are available at the GenBank, assigned an accession number PRJNA417037. File S5 contains plant height phenotypic data used for GWAS analysis. File S6 contains full details of the Materials and Methods employed in this study.

**REFERENCES:**

Bates, D., M. Maechler, and B. Bolker., 2015 Fitting Linear Mixed-Effects Models Using lme4. J. Stat. Softw. 67: 1–48.

Bradbury, P. J., Z. Zhang, D. E. Kroon, T. M. Casstevens, Y. Ramdoss *et al.*, 2007 TASSEL: Software for association mapping of complex traits in diverse samples. Bioinformatics 23: 2633–2635.

Elshire, R. J., J. C. Glaubitz, Q. Sun, J. a Poland, K. Kawamoto *et al.*, 2011 A robust, simple genotyping-by-sequencing (GBS) approach for high diversity species. PLoS One 6: e19379.

Matthies, I. E., M. Malosetti, M. S. Röder, and F. V Eeuwijk, 2014 Genome-wide association mapping for kernel and malting quality traits using historical European barley records. PLoS One 9: e110046.

R Core Team, 2014 *R: A language and environment for statistical computing*. R Foundation for Statistical Computing, Vienna, Austria.

Wickham, H., 2009 *ggplot2: Elegant graphics for data analysis* (G. Robert, H. Kurt, & P. Giovanni, Eds.). Springer, New York, NY.

Wimmer, V., T. Albrecht, H.-J. Auinger, and C.-C. Schön, 2012 Synbreed: A framework for the analysis of genomic prediction data using R. Bioinformatics 28: 2086–2087.

Zhang, D., J. Li, R. O. Compton, J. Robertson, V. H. Goff *et al.*, 2015 Comparative genetics of seed size traits in divergent cereal lineages represented by sorghum (Panicoidae) and rice (Oryzoidae). G3 5: 1117–1128.
